# Polar metabolomics in human muscle biopsies using a liquid-liquid extraction and full-scan LC-MS

**DOI:** 10.1016/j.xpro.2022.101302

**Published:** 2022-04-16

**Authors:** Bauke V. Schomakers, Jill Hermans, Yorrick R.J. Jaspers, Gajja Salomons, Frédéric M. Vaz, Michel van Weeghel, Riekelt H. Houtkooper

**Affiliations:** 1 Laboratory Genetic Metabolic Diseases, Amsterdam UMC, Location AMC, Meibergdreef 9, AZ 1105 Amsterdam, the Netherlands; 2 Core Facility Metabolomics, Amsterdam UMC, Location AMC, Meibergdreef 9, AZ 1105 Amsterdam, the Netherlands

**Keywords:** Health Sciences, Mass Spectrometry, Metabolism, Metabolomics

## Abstract

We describe here a user-friendly analysis protocol for semi-targeted polar metabolomics in human muscle biopsies using Zwitterionic Hydrophilic Interaction Liquid Chromatography and high-resolution full-scan mass spectrometry. Previously, this protocol has been used for *Caenorhabditis elegans*. Here we show that it can be successfully applied to human muscle biopsies with minor adjustments. Summarized instructions for other matrices are also provided. As peak integration in metabolomics can be challenging, we provide expected retention times and extensive peak descriptions to aid this process.

For complete details on the use and execution of this protocol, please refer to Molenaars et al. (2021).

## Highlights

• Protocol for polar metabolomics using liquid-liquid extraction and full-scan MS
• Semiquantitative analysis of 150 metabolites in human muscle biopsies
• Full peak integration and data processing instructions
• Additional instructions on 32 more biological matrices provided

## Graphical abstract

**Figure:**
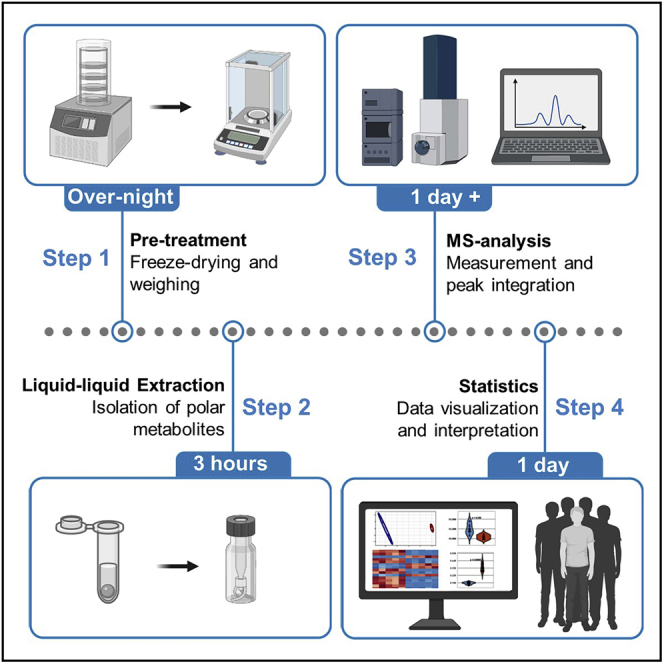

## Before you begin

The protocol below describes the procedure for polar metabolomics in human muscle biopsies (Janssens et al., 2022), but the same protocol has been successfully applied for human (Remie et al., 2021; Connell et al., 2021; Remie et al., 2020), mouse (van der Stelt et al., 2021; Zapata-Pérez et al., 2021) or rat tissues (brown adipose tissue, white adipose tissue, heart, liver, kidney, brain, placenta, arterial plaque), cultured cells (primary skin fibroblasts, Human Embryonic Kidney (HEK) cells, bronchial epithelial cells, cumulus cells, oocytes, organoids), blood samples (Zapata-Pérez et al., 2021) (serum, plasma, erythrocytes, platelets, peripheral blood mononuclear cells, whole blood, macrophages), other bodily fluids (saliva, sweat, urine, fecal water, bile, cerebrospinal fluid), model organisms (Molenaars et al., 2021; Liu et al., 2020; Gao et al., 2018; Molenaars et al., 2020) (yeast, fruit flies, zebrafish and C. elegans) and culture medium. Additional details on these matrices can be found in Table S1 Sample Matrix Overview.

### Institutional permissions

Human muscle biopsies were obtained as part of a previous study (Grevendonk et al., 2021; Janssens et al., 2022). Research was approved by the institutional medical ethical committee, and experiments were conducted in agreement with the Declaration of Helsinki. All participants provided written informed consent. The study was registered at clinicaltrials.gov with identifier NCT03666013. Data obtained for this study, using this protocol, has been published in Nature Aging by Janssens et al. (2022).

### Preparing the samples for extraction

**Timing: 8 h**

**Timing: 3 min per sample**

During this step, wet muscle tissue is freeze-dried. This enables correction of data using dry weight.

***Note:*** Make sure to snap-freeze muscle biopsies immediately after isolation using liquid nitrogen, before storage at −80°C to preserve sensitive metabolites. The tissues need to remain frozen until they go into the freeze-drying machine (*e.g*., a Zirbus Technology VaCo 2).

1. Freeze-dry 10–15 mg of wet muscle tissue.
  a. Pre-cool the freeze-dryer.
  b. Either loosen the lid of each sample tube slightly or poke holes in the top of the tube using a needle.
  c. Place the tube containing the muscle tissue in the freeze-dryer.
  d. Use an 8 h program (e.g., 0.05 bar and −50°C) to freeze-dry the tissues.

**CRITICAL:** When ready, open the valve to break the vacuum slowly, as not to eject any freeze-dried tissue from the tubes as air flows back in.

**CRITICAL:** If the freeze-drying step is unsuccessful, samples will have spent an entire night out of the freezer and are likely not suitable for analysis, with little options for troubleshooting. Double check that each tube has at least a slight opening (Unscrew the cap slightly or poke holes in the top) and your machine settings. Additionally, make sure the freeze-dryer reaches the appropriate temperature and pressure.

***Note:*** To avoid overloading the analytical system and to make comparative analysis between samples easier, fixed amounts of freeze-dried tissue are prepared for extraction.

***Note:*** A standard analytical balance can be inaccurate in the range of the weights described here for freeze-dried tissue. If this is an issue, an alternative is to use the balance for an approximate measurement. Then, dry the protein pellet after the extraction and perform a BCA analysis on it, for instance using a Pierce BCA Protein Assay. This will yield a protein concentration that can also be used for corrections.

**CRITICAL:** It is important to use a safe-lock tube. The sample will be lysed in methanol using a TissueLyzer, and regular tubes have a risk of opening due to the forces exerted on the tube during the homogenization procedure.

**Figure 1:**
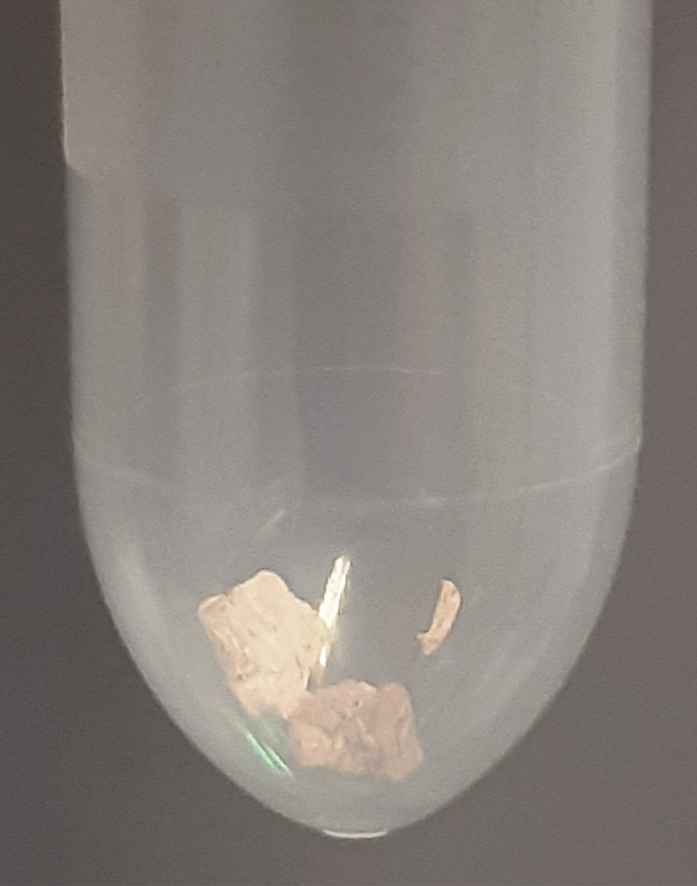
Eppendorf™ tube containing approximately 3 mg of Freeze-dried muscle tissue Freeze-dried muscle tissue is typically beige colored and very tough and flaky material. Quantity of material shown here can be used as a visual reference.

2. Transfer 2–5 mg (aim for 3 mg) of freeze-dried muscle tissue into a clean 2 mL Eppendorf™ Safe-lock tube.
  a. Place the clean 2 mL Eppendorf™ Safe-lock tube on the analytical scale and press Tare.
  b. Use a small spatula to push down and twist on the muscle tissue to break it into smaller pieces and homogenize as much as possible.
  c. Sweep these smaller pieces into the clean 2 mL Eppendorf™ Safe-lock tube using the spatula.
    ***Alternatives:*** Lightly tap the tube containing the tissue above the clean tube until you have transferred enough tissue.
  d. Close the tube.
  e. Clean the outside of the tube with a paper towel before recording the weight. Sample should resemble Figure 1.
    **CRITICAL:** Freeze-dried muscle tissue can be statically charged, causing it to “jump”. Be aware of tissue sticking to the outside of your tube. Make sure the weight you record corresponds to tissue that is actually in the tube.
    **CRITICAL:** Muscle tissue specimens are to be considered biohazardous, and appropriate personal protection equipment (PPE) and institutional biosafety protocols should be observed.

### Preparing internal standard mixture

**Timing: 2 h**

Internal standards are added to aid relative quantitation and are used to correct for sample loss during preparation, ion suppression, or fluctuations in signal in the MS over time.

3. Making a master mix of internal standards.
  a. Using a pipette, add all components in the Internal standard mixture table under material and methods to a large glass vial.
  b. Store at −20°C for a maximum of 6 months.
    **CRITICAL:** Be sure to make plenty of the master mix. If you spill some and have to switch mixtures halfway through the experiment, this will add to the variability of the results and may even result in an unsuccessful experiment.
    **CRITICAL:** This mixture is added to your samples. Be sure to use clean tubes and tools for each standard as well as to make the mixture. Any contamination from cleaning products or biological materials may interfere with your analysis.
    **CRITICAL:** It is possible to leave out certain internal standards or add others depending on your experimental question. However, always test new additions for interferences with endogenous metabolites. When leaving out an internal standard, always make sure there is still adequate coverage of the entire retention time range and chemical diversity of the metabolome.

### Preparing sample solvent

**Timing: 5 min**

Sample solvent is used to dissolve the samples during the final step of preparation. You will need 100 μL per sample. Use only MS grade solvents throughout this protocol.

4. Mixing methanol and Milli-Q water in a 3:2 ratio (v/v).
  a. Add 1.5 mL of methanol for each 1 mL of Milli-Q water. (e.g., 15 mL of methanol and 10 mL of Milli-Q water). Make a fresh solution before every project.

**CRITICAL:** Perform these steps using PPE (e.g., gloves and goggles, depending on institutional guidelines), and in a fume hood as methanol vapors are harmful.

### Preparing liquid chromatography solutions

**Timing: 30 min**

These solvents are used as eluents during the LC-MS method; use only MS-grade solvents. Solvent volumes are suggestions. Typically, solvent B is depleted roughly twice as fast as solvent A.

5. Preparing a 100 mM ammonium acetate solution.
  a. Add 3.85 g ammonium acetate to 500 mL Milli-Q water.
  b. Swirl until fully dissolved.
  c. Store at 4°C for a maximum of six months.
6. Preparing Eluent A: Milli-Q water:acetonitrile 90:10 containing 5 mM ammonium acetate.
  a. Add 850 mL of Milli-Q water to a clean 1 L eluent bottle.
  b. Add a 50 mL volume of the 100 mM ammonium acetate solution created in step 5.
  c. Add 100 mL of acetonitrile.
  d. Cap the bottle and swirl until well-mixed.
  e. Degas the solution depending on the type of chromatographic system.
  f. Store at 20°C for a maximum of six months.
7. Preparing Eluent B: Milli-Q water:acetonitrile 10:90 containing 5 mM ammonium acetate.
  a. Add 100 mL of Milli-Q water to a clean 2 L eluent bottle.
  b. Add a 100 mL volume of the 100 mM ammonium acetate solution created in step 5.
  c. Add 1,800 mL of acetonitrile.
  d. Cap the bottle and swirl until well-mixed.
  e. Degas the mobile phase solution depending on the type of chromatographic system.
  f. Store at 20°C for a maximum of six months.

**CRITICAL:** Perform these steps using PPE (e.g., gloves and goggles, depending on institutional guidelines), and in a fume hood as acetonitrile vapors are harmful.

**CRITICAL:** Make sure your glassware is clean and does not contain contaminants. Certain cleaning agents appear to contain metabolites such as succinate, which is a TCA-cycle intermediate, detected with this technique. Using empty MS-grade acetonitrile bottles as eluent bottles is advised, as these are as clean as the solvents by definition, or rinse your glassware with suitable solvents before use.

### Pre-heating the vacuum concentrator

**Timing: 20 min**

8. Pre-heat the vacuum concentrator (e.g., Genevac™ miVac Centrifugal Concentrator) to 60°C using the pre-heat function.

## Key resources table

**Table:** 

REAGENT or RESOURCE	SOURCE	IDENTIFIER
**Biological samples**		

Human Muscle Biopsies	Joris Hoeks (Grevendonk et al., 2021; Janssens et al., 2022)	n/a

**Chemicals, peptides, and recombinant proteins**		

Lactic acid-D_3_	MilliporeSigma (sigmaaldrich.com)	Cat#616702
^13^C_3_-Pyruvate	MilliporeSigma (sigmaaldrich.com)	Cat#490717
^13^C_6_-D-Glucose	MilliporeSigma (sigmaaldrich.com)	Cat#389374
^13^C_6_-D-Glucose--6-phosphate	Cambridge Isotope Laboratories, inc (isotope.com)	Cat#CLM-8367
^13^C_6_-D-Fructose-1,6-diphosphate	Cambridge Isotope Laboratories, inc (isotope.com)	Cat#CLM-8962
Adenosine-^15^N_5_-triphosphate	MilliporeSigma (sigmaaldrich.com)	Cat#707783
Guanosine-^15^N_5_-triphosphate	MilliporeSigma (sigmaaldrich.com)	Cat#707775
Adenosine-^15^N_5_-monophosphate	MilliporeSigma (sigmaaldrich.com)	Cat#662658
Guanosine-^15^N_5_-monophosphate	MilliporeSigma (sigmaaldrich.com)	Cat#662674
Succinic acid-D_6_	MilliporeSigma (sigmaaldrich.com)	Cat#488356
Citric acid-D_4_	MilliporeSigma (sigmaaldrich.com)	Cat#485438
L-Carnitine-D_3_	MilliporeSigma (sigmaaldrich.com)	Cat#616737
Thymine-D_4_	MilliporeSigma (sigmaaldrich.com)	Cat#487066
^13^C_2_-Glycine	MilliporeSigma (sigmaaldrich.com)	Cat#283827
Alanine-D_4_	MilliporeSigma (sigmaaldrich.com)	Cat#485845
Arginine-D_7_	MilliporeSigma (sigmaaldrich.com)	Cat#776408
Aspartate-D_3_	MilliporeSigma (sigmaaldrich.com)	Cat#589667
^13^C_1_-Citrulline	Cambridge Isotope Laboratories, inc (isotope.com)	Cat#CLM-4899
Glutamate-D_3_	MilliporeSigma (sigmaaldrich.com)	Cat#749435
Glutamine-D_5_	MilliporeSigma (sigmaaldrich.com)	Cat#616303
^13^C_6_-Isoleucine	MilliporeSigma (sigmaaldrich.com)	Cat#604798
Leucine-D_3_	MilliporeSigma (sigmaaldrich.com)	Cat#486825
Lysine-D_4_	MilliporeSigma (sigmaaldrich.com)	Cat#489034
Methionine-D_3_	MilliporeSigma (sigmaaldrich.com)	Cat#300616
Ornithine-D_6_	MilliporeSigma (sigmaaldrich.com)	Cat#749443
Phenylalanine-D_5_	Cambridge Isotope Laboratories, inc (isotope.com)	Cat#DLM-1258
Proline-D_7_	Cambridge Isotope Laboratories, inc (isotope.com)	Cat#DLM-487
Serine-D_3_	MilliporeSigma (sigmaaldrich.com)	Cat#688436
Tryptophan-D_5_	MilliporeSigma (sigmaaldrich.com)	Cat#615862
Tyrosine-D_4_	MilliporeSigma (sigmaaldrich.com)	Cat#792721
Valine-D_8_	MilliporeSigma (sigmaaldrich.com)	Cat#486612
Ammonium Acetate	MilliporeSigma (sigmaaldrich.com)	Cat#431311

**Other**		

Merck Millipore SeQuant ZIC-cHILIC column (PEEK 100 × 2.1 mm, 3 μm particle size)	MilliporeSigma (sigmaaldrich.com)	Cat# 1.50657.0001
Zirbus Technology VaCo 2	Zirbus (zirbus.nl)	N/A
Eppendorf® Combitips Advanced	MilliporeSigma (sigmaaldrich.com)	Cat#EP0030089464-100EA
Eppendorf Safe-Lock Tubes, 2.0 mL, Eppendorf Quality^TM^, colorless, 1,000 tubes	Eppendorf (online-shop.oppendorf.com)	Cat#0030120205
Qiagen Stainless Steel Beads, 5 mm (200)	Fisher Scientific (fishersci.com)	Cat#69989
Qiagen TissueLyser II	QIAGEN (qiagen.com)	Cat#85300
Multi-tube vortex shaker, analogue, VXMTAL	VWR (vwr.com)	Cat#OHAU30392166
Eppendorf™ Refrigerated Centrifuge	Fisher Scientific (fishersci.com)	Cat#5425R
Genevac™ miVac Centrifugal Concentrators	Fisher Scientific (fishersci.com)	Cat#44270.75 SEK - 77996.11 SEK
150 μL Conical Glass Insert (total volume 200 μL), 31 × 5 mm, Tip: 15 mm, pk.100	BGB (https://www.bgb-info.com/)	Cat#110500
1.5 mL Crimp Neck Vial 32 × 11.6 mm (clear), narrow opening, pk.100	BGB (https://www.bgb-info.com/)	Cat#110400
Waters Acquity UPLC	Waters (waters.com)	N/A
Ultra-high resolution LC-QTOF MS Impact II	Bruker (bruker.com)	N/A
TASQ® Software (e.g., Version 2021.1.2.452)	Bruker (bruker.com)	N/A

## Materials and equipment

The table below provides all details for reagents added to the internal standard mix. Amounts are for a single sample. The total amount is the amount of this mixture added to each sample. All components are dissolved in Milli-Q water. Store all solutions at −20°C in practical aliquots (e.g., 20 samples worth of internal standard mixture). Unused solutions can be stored indefinitely, but should be analyzed before each project to determine quality.

***Note:*** As sample volumes change between steps in this protocol, reported concentrations of the internal standard mixture often lead to confusion. However, reporting absolute amounts is also impractical. Therefore, the Final concentration indicated in the table below is the concentration present in sample contained in the UPLC autosampler vials, not in the 155 μL internal standard mixture (concentrations in this mixture change with the addition or removal of reagents). For example, 5 μL of 200 μM of Lactic acid-D_3_ solution is added to the internal standard mixture, which is then added to the tissue extract. After the protocol has been performed, the final concentration of Lactic acid-D_3_ in the muscle tissue sample extract that is injected in the LC-MS is 10 μM.

***Note:*** We recommend making an internal standard mix for the whole experiment. Due to pipetting inaccuracies, we advise to always make 10% additional units of the internal standard mixture, with a minimum of 2. For instance, for a series of 20 samples, we add 110 μL of each reagent, for a total of 3.41 mL.

**Table:** Internal standard mixture

Reagent	Final concentration (in 100 μL sample)	Amount
Lactic acid-D_3_ (200 μM)	10 μM	5 μL
^13^C_3_-Pyruvate (100 μM)	5 μM	5 μL
^13^C_6_-D-Glucose (2,000 μM)	100 μM	5 μL
^13^C_6_-D-Glucose-6-phosphate (200 μM)	10 μM	5 μL
^13^C_6_-D-Fructose-1,6-diphosphate (200 μM)	10 μM	5 μL
Adenosine-^15^N_5_-triphosphate (1,000 μM)	50 μM	5 μL
Guanosine-^15^N_5_-triphosphate (1,000 μM)	50 μM	5 μL
Adenosine-^15^N_5_-monophosphate (1,000 μM)	50 μM	5 μL
Guanosine-^15^N_5_-monophosphate (1,000 μM)	50 μM	5 μL
Succinic acid-D_6_ (100 μM)	5 μM	5 μL
Citric acid-D_4_ (100 μM)	5 μM	5 μL
L-Carnitine-D_3_ (100 μM)	5 μM	5 μL
Thymine-D_4_ (200 μM)	10 μM	5 μL
^13^C_2_-Glycine (10,000 μM)	500 μM	5 μL
Alanine-D_4_ (250 μM)	12.5 μM	5 μL
Arginine-D_7_ (250 μM)	12.5 μM	5 μL
Aspartate-D_3_ (250 μM)	12.5 μM	5 μL
^13^C_1_-Citrulline (250 μM)	12.5 μM	5 μL
Glutamate-D_3_ (250 μM)	12.5 μM	5 μL
Glutamine-D_5_ (250 μM)	12.5 μM	5 μL
^13^C_6_-Isoleucine (250 μM)	12.5 μM	5 μL
Leucine-D_3_ (250 μM)	12.5 μM	5 μL
Lysine-D_4_ (250 μM)	12.5 μM	5 μL
Methionine-D_3_ (250 μM)	12.5 μM	5 μL
Ornithine-D_6_ (250 μM)	12.5 μM	5 μL
Phenylalanine-D_5_ (250 μM)	12.5 μM	5 μL
Proline-D_7_ (250 μM)	12.5 μM	5 μL
Serine-D_3_ (250 μM)	12.5 μM	5 μL
Tryptophan-D_5_ (250 μM)	12.5 μM	5 μL
Tyrosine-D_4_ (250 μM)	12.5 μM	5 μL
Valine-D_8_ (250 μM)	12.5 μM	5 μL
**Total volume of internal standard to be added to each sample**	**n/a**	**155 μL**

Store this solution at −20°C.

***Alternatives:*** The above list of internal standards corresponds to a diverse range of endogenous metabolites, with a good coverage of the entire retention time of the method. We have purchased our standards at Millipore-Sigma and Cambridge Isotope Laboratories, Inc. However, depending on your research question it is possible to add more or leave some internal standard compounds out depending on availability and convenience. In general, it is advised to correct for internal standards with similar retention times and chemical structures. Although there are options from various vendors, for our procedures we used the equipment listed in the key resources table.

**Table:** Eluent A

Reagent	Final concentration	Amount
Milli-Q water	n/a	850 mL
Ammonium acetate solution (100 mM)	5 mM	50 mL
Acetonitrile	n/a	100 mL
**Total**	**n/a**	**1,000 mL**

Store at 20°C.

**Table:** Eluent B

Reagent	Final concentration	Amount
Milli-Q water	n/a	100 mL
Ammonium acetate solution (100 mM)	5 mM	100 mL
Acetonitrile	n/a	1,800 mL
**Total**	**n/a**	**2,000 mL**

Store at 20°C.

## Step-by-step method details

### Breakdown of tissue

**Timing: 30 min**

During this step, a TissueLyser is used to break down the major biological organization of the muscle biopsies. Try to work in a fume hood as much as possible due to possible methanol vapors. Additionally, keep samples on ice to limit the possible breakdown of more unstable metabolites.

**Figure 2:**
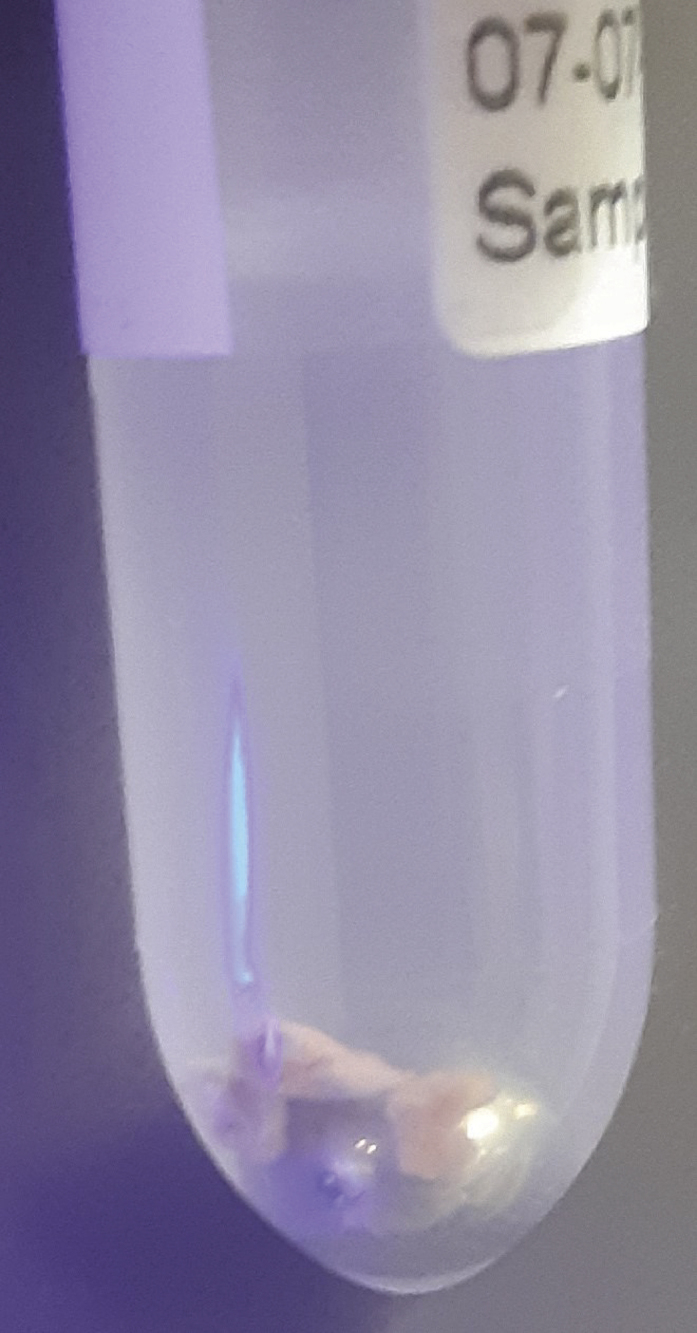
Sample after the addition of methanol, Milli-Q water, and internal standard mixture and a stainless steel bead After the addition of these components, the tissue typically stays intact; *e.g*., no coloration of the solvents, no dissociation of the tissue itself.

**Figure 3:**
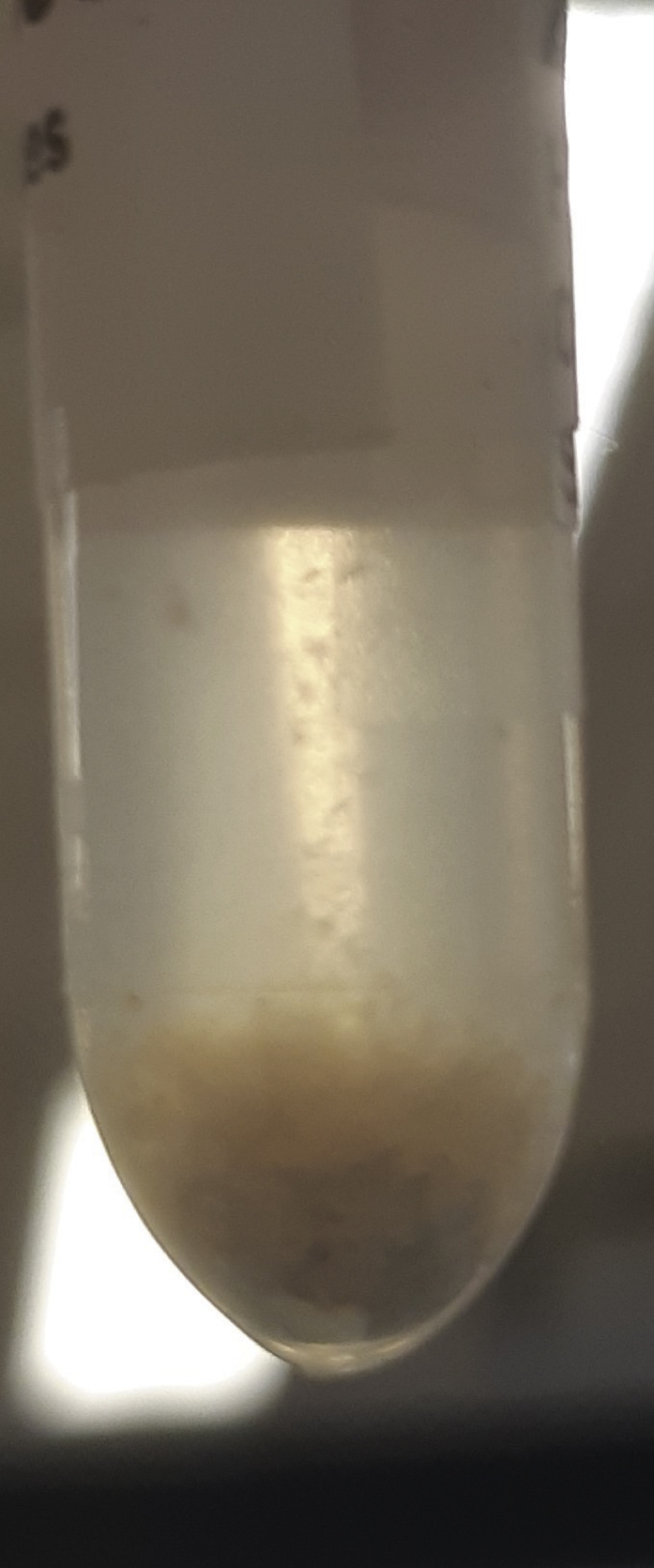
Sample after using the TissueLyser After using the TissueLyser, sample solvents typically look cloudy, and the tissue itself should have lost its flaky structure, instead forming a puffy goo at the bottom of the tube.

***Note:*** In our TissueLyser, position of the sample in the machine influences the distance each sample travels in the arm as the machine is working. To ensure every sample has been treated equally, we tend to reverse the sample position in the sample trays halfway through the procedure.

1. To each sample, add the following components in order.
  **CRITICAL:** Use only MS-grade solvents throughout this protocol.
  **CRITICAL:** When opening the tubes to add these reagents, statically charged tissue may sometimes jump out of the tube or into the lid. Make sure to only open one tube at a time to protect the other samples and be careful not to lose any material.
  ***Note:*** Transferring pure methanol and chloroform accurately can be tricky due to their viscosity. Prime the pipette tip by pipetting these liquids up and down three times before moving the final volume. Positive displacement pipettes can also provide a solution (e.g., Eppendorf® Combitips Advanced).
  a. 500 μL of methanol. (always add this before adding Milli-Q water).
  b. 155 μL of internal standard mixture.
  c. 345 μL of Milli-Q water.
  d. Gently roll a 5 mm stainless steel bead (e.g., Qiagen Stainless Steel Beads, 5 mm) into the tube. Sample should now resemble Figure 2.
    **CRITICAL:** Solvent ratios during the liquid-liquid extractions are important. Methanol and chloroform are miscible, as are methanol and water. It is the 2:1:1 chloroform:methanol:water (v/v/v) ratio that results in a two-phase system. If, due to human error, this ratio gets disturbed, read the troubleshooting section.
    **CRITICAL:** Muscle tissues specimens are to be considered biohazardous, and appropriate personal protection equipment (PPE) and institutional biosafety protocols should be observed.
    **CRITICAL:** Methanol vapor is harmful. Wear PPE (e.g., gloves and goggles, depending on institutional guidelines), and work in a fume hood.
    **CRITICAL:** Do not drop the stainless steel bead into the tube carelessly. Sample may splash out. The bead may also be added before the solvents, but we have found this to be more difficult, due to static effects.
    **CRITICAL:** Keep in mind that the bead comes into direct contact with your sample. Do not touch the beads with your bare hands and make sure the beads are clean before use. Check with your supplier if the beads you use require a solvent wash before use.
    ***Note:*** It is advised to perform a blank extraction as a control to aid peak identification at later stages. The extraction of the control blank sample contains all components, including internal standards, and goes through all steps, but does not contain muscle tissue.

2. Use the TissueLyser (e.g., Qiagen TissueLyser II) to break down the tissue for 5 min at 30 times/s. Visually inspect each sample to look like Figure 3. Repeat this step if necessary, until all tissues look similar to Figure 3.

### Liquid-liquid extraction and isolation of the polar layer

**Timing: 2 h**

During this step, further breakdown of biological organization and removal of lipophilic compounds is accomplished through the addition of chloroform. The polar layer of the liquid-liquid extraction is isolated and concentrated.

**CRITICAL:** Methanol and chloroform vapors are harmful. Wear PPE (e.g., gloves and goggles, depending on institutional guidelines), and work in a fume hood as much as possible due to possible methanol and chloroform vapors. Additionally, practically useful laboratory gloves (such as nitrile gloves) provide little protection against chloroform spillage. It is strongly advised to read the Material Safety Data Sheet (MSDS) of chloroform before use.

**Figure 4:**
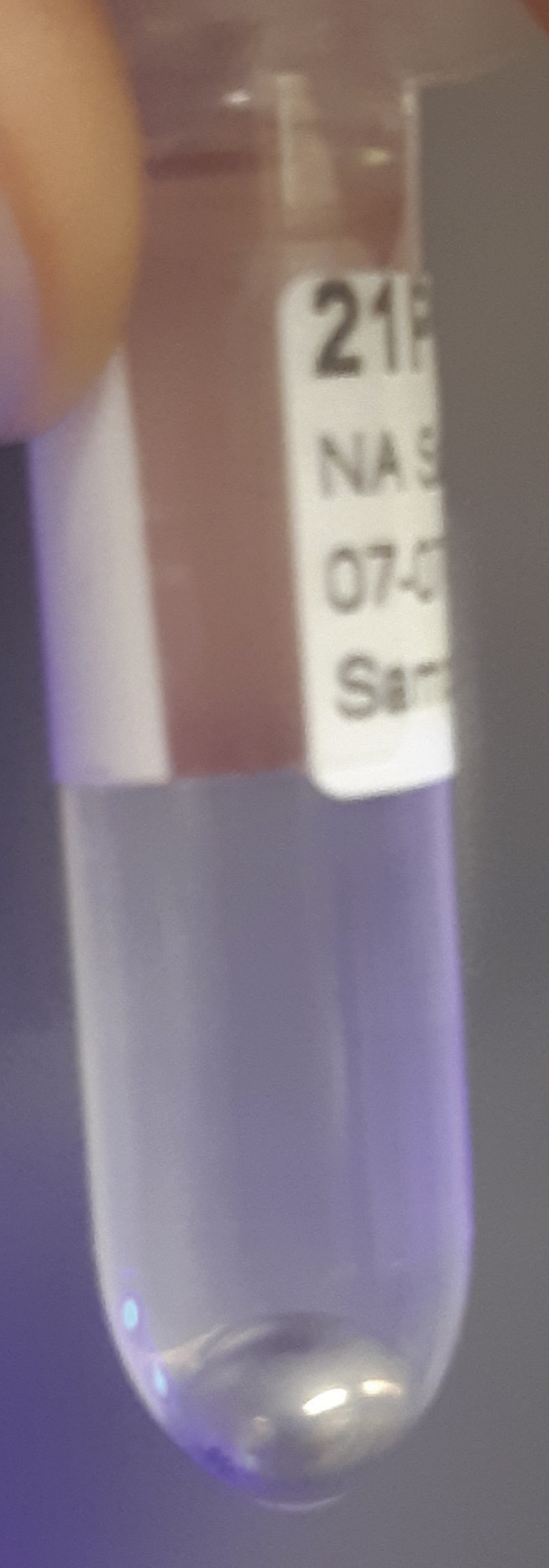
Sample after the addition of chloroform After the addition of chloroform, which will form the bottom phase as it is the heaviest solvent in the extraction, the polar top phase typically takes on the color of the muscle tissue.

**Figure 5:**
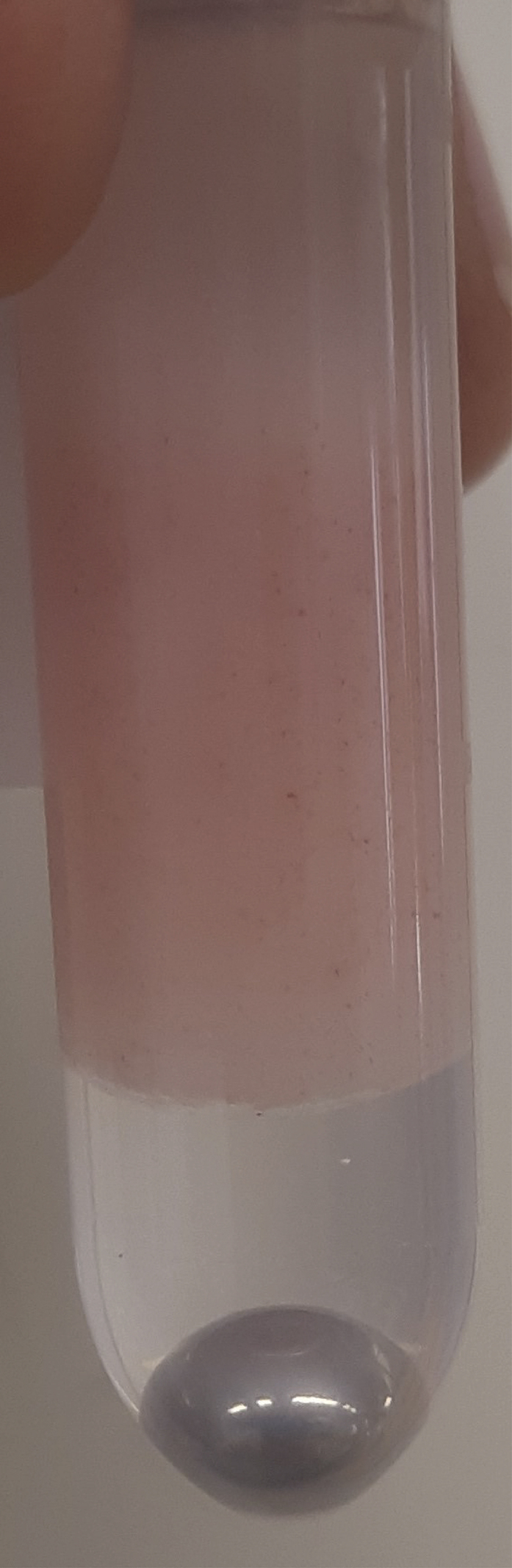
Sample after thorough mixing After mixing, the top phase appears to be larger than the bottom phase. This is normal and samples will revert back to a 50:50 polar:apolar solvent distribution after centrifugation.

**Figure 6:**
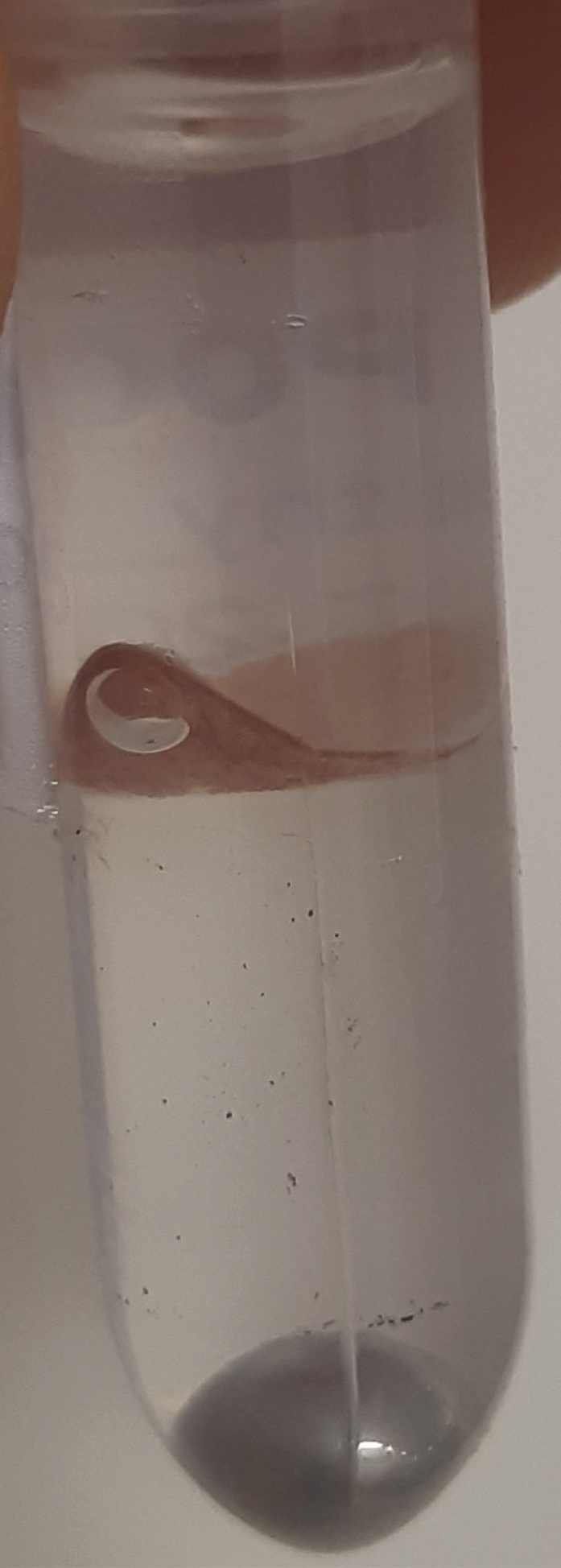
Sample after centrifugation After centrifugation, three phases will be visible. A polar top phase, an apolar bottom phase and a protein pellet in between both of those phases.

3. Creating a two-phase system.
  a. Add 1,000 μL of chloroform to the homogenized sample. After addition, samples should resemble Figure 4.
  b. Using a vortex or multi-tube vortexer (e.g., Multi-tube vortex shaker, analogue, VXMTAL), thoroughly mix each sample for 2 min. After mixing, samples should resemble Figure 5.
  c. Centrifuge samples for 10 min at 20,000 × *g* and 4°C (e.g., using an Eppendorf™ Refrigerated Centrifuge). The heavier chloroform is the lower layer, while the top layer is a mixture of water and methanol. In between are precipitated proteins. The top and bottom layers should be roughly of equal size, resembling Figure 6.

**CRITICAL:** Occasionally, chloroform vapor escapes the Eppendorf™ tubes in the centrifuge. Be sure to place the centrifuge in a fume hood or use other effective ventilation before performing this part of the experiment.

**Figure 7:**
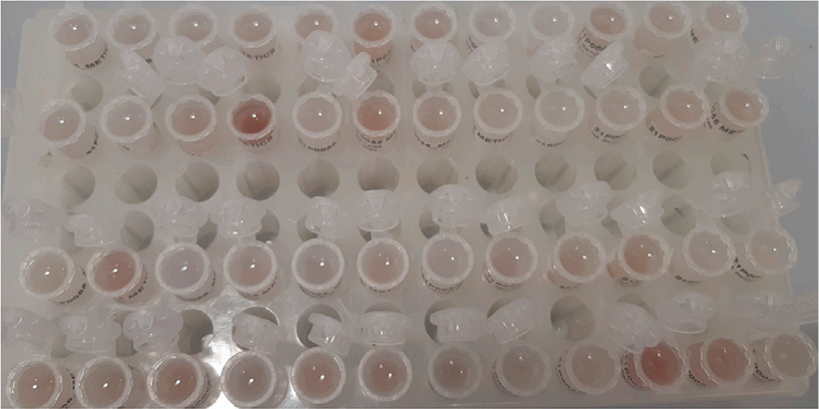
An overview of multiple liquid-liquid extractions of muscle tissues Color differences between samples can be quite noticeable; this is to be expected.

***Note:*** Samples can differ quite a bit in color, as shown in Figure 7, for instance due to blood being present in the samples. This should not alarm you, but it can be useful to make a note of this in your lab journal.

**Figure 8:**
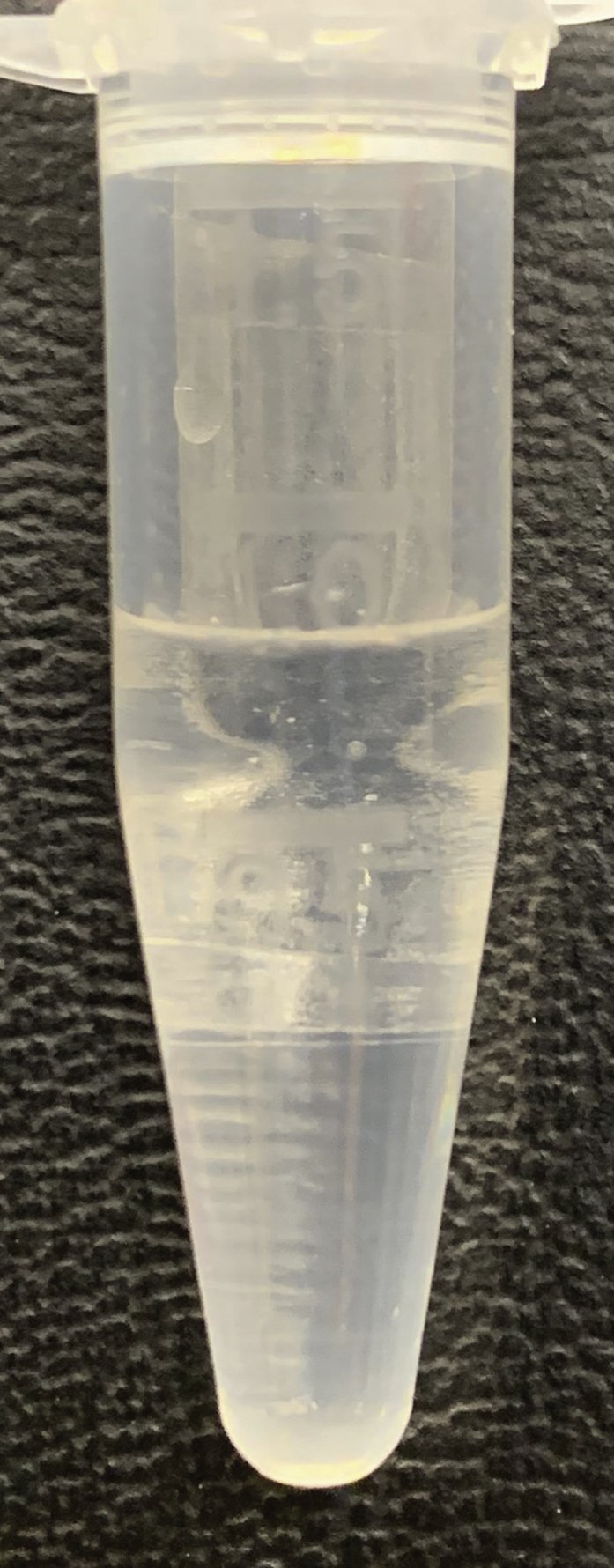
Isolated polar phase of the liquid-liquid extraction The isolated polar phase should be a clear liquid, without any visible residual protein.

**Figure 9:**
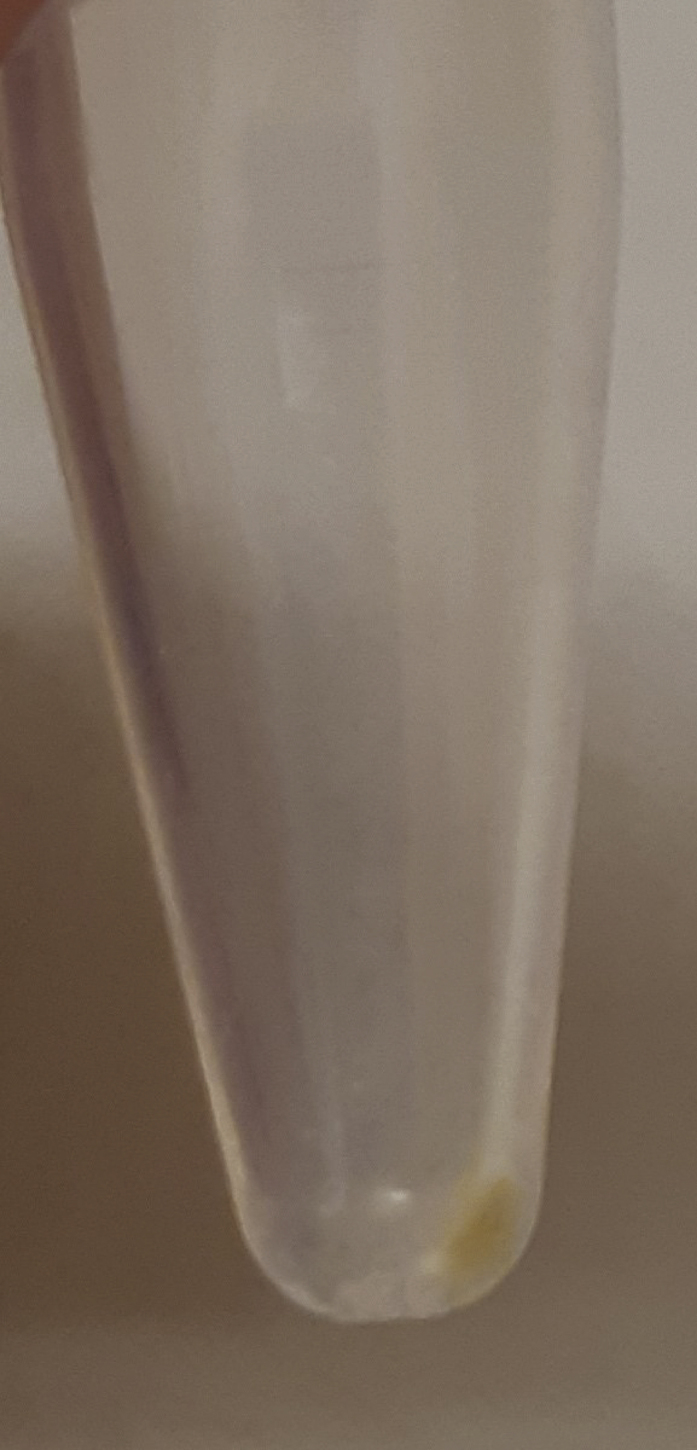
Sample after evaporation After evaporation of the polar solvents using a vacuum concentrator, a yellow(ish) pellet is typically visible. Additionally, white streaks can be visible on the side of the tube (not shown here).

4. Isolating the top layer of the extraction for further preparation.
  a. Using a 1 mL pipette, carefully transfer approximately 850 μL of the top layer to a clean 1.5 mL Eppendorf™ tube. The transferred phase should be a clear liquid without debris, as shown in Figure 8.
    **CRITICAL:** The easiest way to perform this step is by holding the tube in one hand, and pipetting with the other. Be aware that the hand that holds the tube can be exposed to spillage, so wear a glove on whichever hand you use to hold the tubes and work in a fume hood. Keep in mind that it is important not to disturb the chloroform layer during this step. Both from an analytical and safety perspective.
    ***Note:*** If the protein pellet is seriously disturbed during isolation, repeat step 3c. If the pellet is flaky, try to avoid it as much as possible. There is a later step that gets rid of residual protein in the mixture.

5. Concentrating the samples.
  a. Place the 1.5 mL Eppendorf™ tubes in the vacuum concentrator.
  b. Run the pre-heated vacuum concentrator for 1.5 h at 60°C, or until samples are dry. A yellowish to white residue is typically visible, as shown in Figure 9, with circular white streaks above it.

**CRITICAL:** Be aware that methanol has a boiling point of 65°C and the vacuum concentrator is heated to 60°C, causing almost instant evaporation as samples are placed in the machine. As methanol makes up half of the solvent volume and fumes are very harmful, it is strongly advised to perform this step in a fume hood.

### Resuspension and transfer to UPLC autosampler vials

**Timing: 30 min**

During this step, metabolites are reconstituted in a solution that is best suited for the UPLC-mass spectrometry analysis, and any residual protein and other insoluble components are removed.

**Figure 10:**
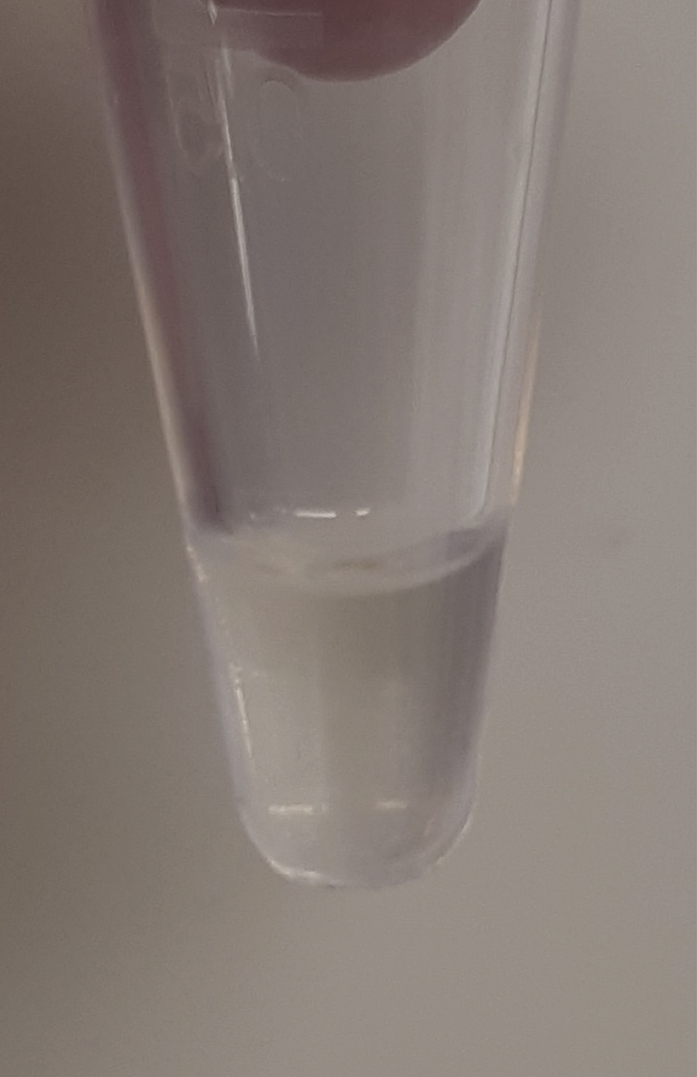
Sample after reconstitution After the addition of 3:2 methanol:Milli-Q water, followed by thorough mixing and centrifugation, samples should resemble a clear liquid. It is possible for a tiny pellet to be present.

6. Resuspension of the samples.
  a. Add 100 μL of 3:2 methanol:Milli-Q water (v/v) to each sample in their 1.5 mL Eppendorf™ tube.
  b. Using a vortex or multi- tube vortexer, thoroughly mix each sample for 2 min. Samples should be a non-turbid, clear liquid, as shown in Figure 10.
  c. Centrifuge samples for 10 min at 20,000 × *g* and 4°C.

***Note:*** Place each 1.5 mL Eppendorf™ tube in the centrifuge facing the same direction. This way, any pellet will form in a predictable spot and can be more easily avoided in further steps.

**Figure 11:**
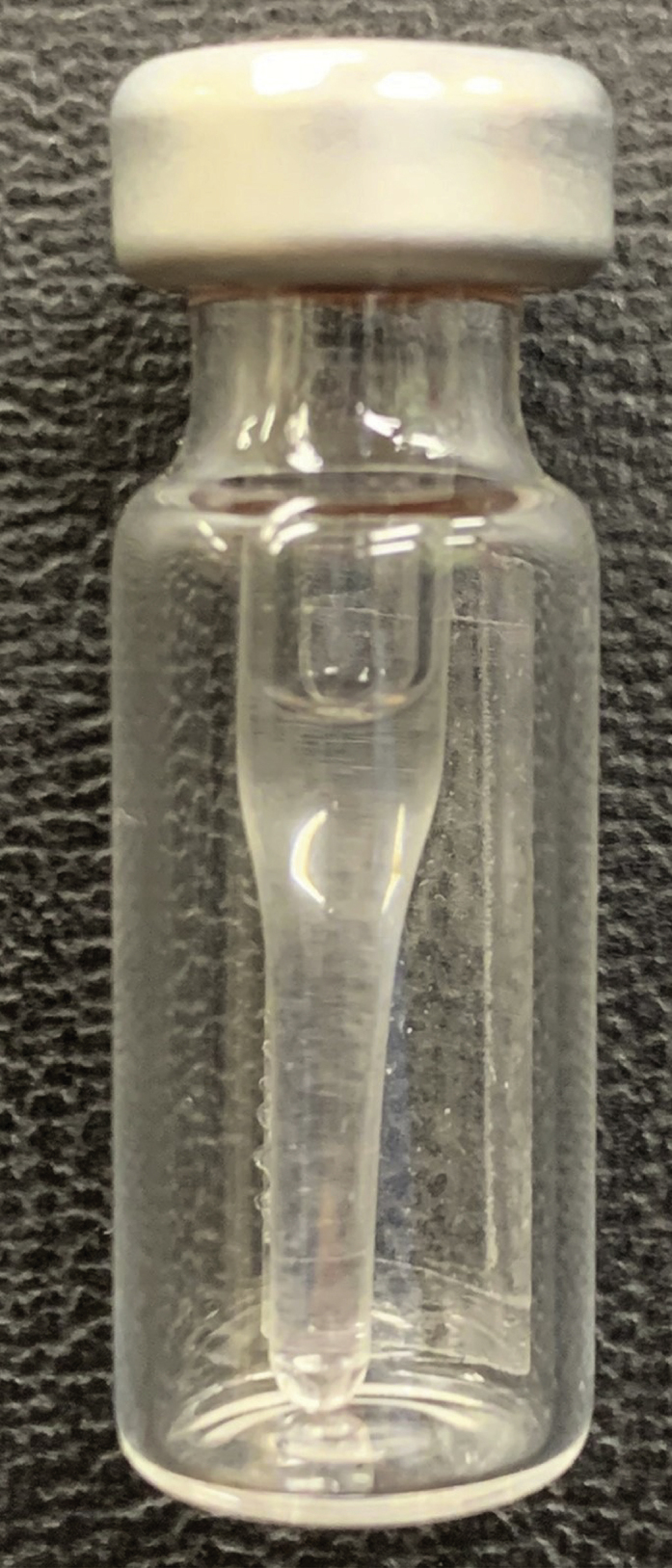
Sample after transfer to a UPLC autosampler vial Samples should be completely clear of any debris. Always check the bottom of the vial insert for any solid matter. Any color previously held by the sample is typically not observed in these small volumes. Slightly discolored samples can still be injected, as long as they do not contain any debris or cloudiness.

7. Transferring supernatant to UPLC autosampler vials.
  **CRITICAL:** This is the final clean-up step before injection into the UPLC and it is therefore important to be very careful not to transfer any protein or other debris.
  a. Using a pipette, carefully transfer approximately, but no more than, an 80 μL volume of supernatant from each sample to clean UPLC autosampler vials (e.g., BGB 1.5 mL Crimp Neck Vial, with a BGB 150 μL Conical Glass Insert), avoiding the pellet. If the pellet is disturbed, repeat step 6c. Samples should resemble Figure 11.
    **CRITICAL:** Save the 1.5 mL Eppendorf™ tubes for the next step! Do not throw them away.

### Making a pooled sample

**Timing: 5 min**

During this step, a pooled sample is created that can be used to check analytical robustness.

**Figure 12:**
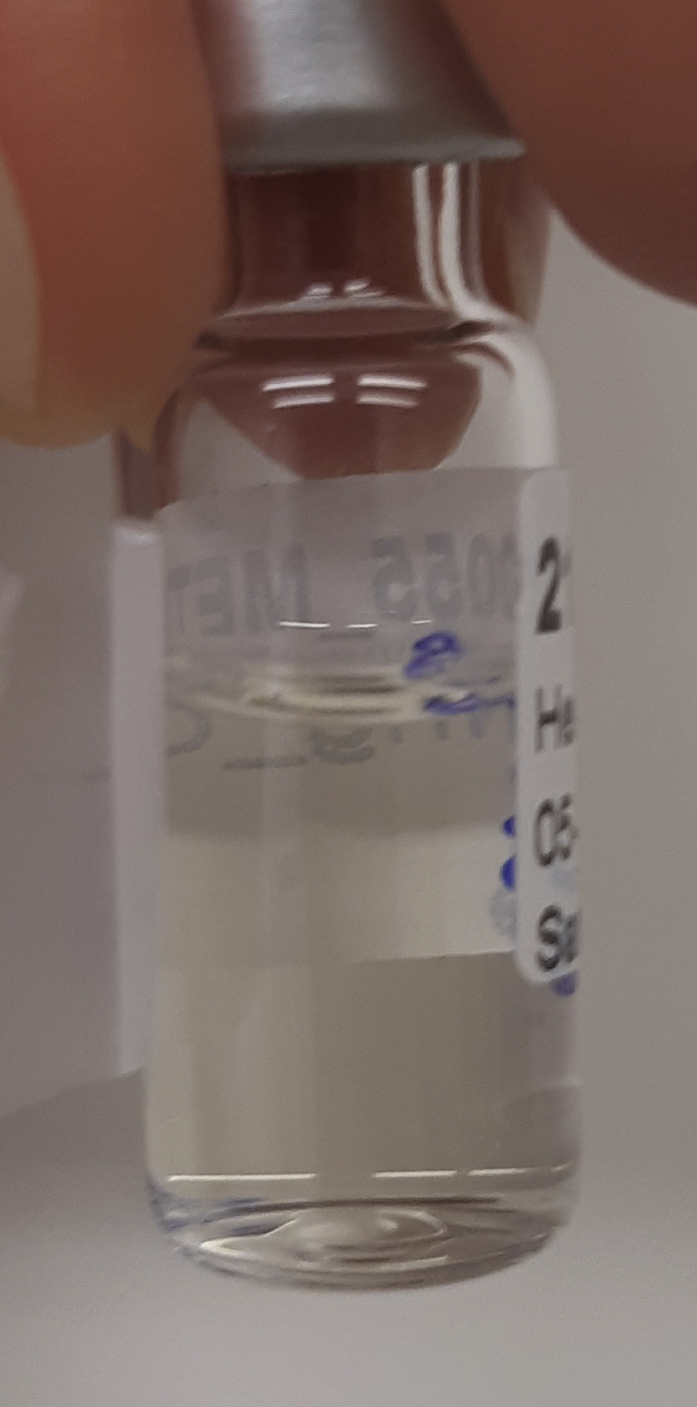
Pooled sample for QC measurements Sample should be completely clear of any debris. When working with larger volumes like the example given here, samples tend to still show some coloration; this is expected.

8. Pooling the samples.
  a. Depending on the number of total samples and the size of the pellet in the 1.5 mL Eppendorf™ tube, pipette an amount of supernatant from each sample into a single clean UPLC autosampler vial. Typically, it is possible to take around a 5–10 μL volume from each sample – be sure to vortex-mix the final sample thoroughly. A Pooled sample example is shown in Figure 12.
    **CRITICAL:** This is the final clean-up step before injection into the UPLC and it is therefore important to be very careful not to transfer any protein flakes or other debris.
    **CRITICAL:** Make sure there is at least an 80 μL volume of pooled sample available for analysis.

### Liquid chromatography/mass spectrometry

**Timing: 21 min per sample**

During this step, chromatographic separation of analytes and full-scan MS analysis is achieved.

9. Sample table.
  a. Randomize your samples in the injection sequence before measuring to ensure observed metabolic effects are not analytical artifacts. For instance by using the “=rand()” function in excel and order samples according to the random numbers.
    ***Note:*** The goal is to break any pattern in your samples. Be aware that sometimes a random list can still contain problematic patterns. For instance, measuring several samples from a single sample group in a row. Always check your injection orders and adjust if necessary.
  b. Include an injection of the pooled sample - prepared in step 8 of the step-by-step method details *section -* every 10 samples to check and possibly correct for system stability. We advise measuring this sample in duplicate at the start and at the end of the analysis to be able to check for carry-over effects. This sample can also be used at the start of the sequence to equilibrate the column, for instance by injecting the pooled sample twice.
  c. Include an injection of a blank sample consisting of the 3:2 methanol:Milli-Q water (v/v) solvent used to dissolve each sample.
  d. Include an injection of the blank extraction control blank sample, which contains only internal standards and has progressed through the entire liquid-liquid extraction.

10. Chromatographic separation.
  a. If possible, samples should be chilled at 12°C in the autosampler tray during sample analysis to preserve more sensitive metabolites as much as possible.
  b. Inject a 5 μL volume of each sample onto a Millipore-Sigma SeQuant ZIC-cHILIC column (PEEK 100 × 2.1 mm, 3 μm particle size), with a column heating temperature of 30°C.
  c. Separate the metabolites with the following linear LC gradient, using for instance a Waters Acquity UPLC, and the solvents made in steps 4–6 of the before you begin section at a flow rate of 0.25 mL/min: Dwell at 100% Solvent B, 0–2 min; Ramp to 54% Solvent B at 13.5 min; Ramp to 0% Solvent B at 13.51 min; Dwell at 0% Solvent B, 13.51–19 min; Ramp to 100% B at 19.01 min; Dwell at 100% Solvent B, 19.01–19.5 min.
  d. Equilibrate the column using a 0.4 mL/min flow at 100% B from 19.5-21 min.
    ***Note:*** We have found it practical to keep our UPLC solvents in a reversed phase set-up, even when using ZIC-cHILIC columns, making sure Solvent A is always an aqueous solution and Solvent B is an organic solvent. This makes it easier to flush solvent lines when switching between ZIC-cHILIC and a reversed phase method and we highly recommend this practice. However, it does mean the strong solvent in our method is named Solvent A, going against convention.

11. MS analysis.
  a. Samples should preferably be measured in both positive and negative ionization, using full-scan mode over the range of m/z 50–1200. Additional settings of our Bruker Impact II can be found in Table S2 MS Settings.

## Expected outcomes

Using this protocol, many polar metabolites can be analyzed, resulting in peak areas per metabolite per sample. In Table S3 Integration Details, each peak that can be expected in human muscle biopsies is described. Using these peak areas, comparisons can be made between groups of samples.

## Quantification and statistical analysis

### Peak integration and statistics

**Timing: 10 h**

During this step, peaks are integrated and statistics are done.

1. Peak integration.
  a. Using an appropriate software package for your MS data (e.g., TASQ by Bruker), make *extracted ion chromatograms* for each mass shown in Table S3 Integration Details.
  b. Integrate each visible peak described in Table S3 Integration Details, according to the instructions.
  c. Always compare the peaks of each metabolite to the blank injection, as well as the blank sample containing the internal standards (injected in steps 9c and 9d, under “liquid chromatography/mass spectrometry”). Metabolites with significant intensity in these blanks should be excluded.
    ***Note:*** As this protocol describes the analysis of human muscle, it is possible that for other matrices, additional peaks are visible or can differ slightly from our suggestions here; this can be due to, for instance, dietary effects or inherent tissue differences. Always be aware of identification uncertainties and use additional identification measures such as spiking samples with standards or MS/MS to confirm identifications of critical metabolites.
    ***Note:*** Compare the retention times of metabolites for which there is an internal standard and use these data to adjust our suggested retention times for your own system.
  d. Extract all peak areas from the software for further corrections.

2. Internal standard corrections.
  a. For each sample, divide the peak area of each metabolite by the peak area of an internal standard with comparable chemical properties and retention time. For suggestions on what internal standard to use for each metabolite, see Table S3 Integration Details.

3. Sample weight correction.
  a. Divide all internal-standard-corrected peak areas belonging to a sample by the recorded dry weight of that sample (see step 2e, under “before you begin”). Alternatively, use the protein concentration determined with the Pierce BCA Protein Assay instead of the “Dry weight of muscle tissue” if the analytical balance is not accurate enough.
    $$Corrected\;peak\;area\;=\frac{\left(Raw\;peak\;area\;of\;metabolite/Internal\;s\tan dard\;peak\;area\right)}{\left(Dry\;weight\;of\;muscle\;tissue\right)}$$
    ***Note:*** Sample weight correction is not performed for the pooled samples.

4. Quality control check.
  a. For each metabolite, calculate the average, standard deviation and coefficient of variance using the measurements of your pooled samples. For a coefficient of variance of e.g., >20%, be extra careful with interpretation of your data, especially when differences between groups are minimal.
  b. This pooled sample should not show any trends after corrections for internal standards. It is difficult to add an objective threshold for these types of variables. However, be aware of -for instance- a steady decline or increase in metabolite abundance. Or, repeating patterns in your intensities; e.g., every third sample has a decreased overall abundance. In summary, fluctuations in metabolite abundance in pooled samples should be minor and random.

5. Statistics.
  a. Using the Corrected Peak Area calculated in steps 2 and 3, it is now possible to calculate fold changes between groups for each metabolite, create volcano plots, create heat maps of metabolite intensities, perform principal component analysis between groups, perform pathway analyses for significantly affected metabolites, or other statistical operations.

## Limitations

This method provides reasonable identification and relative quantification of metabolites. However, be aware that this is not a dedicated method for any individual metabolite. In-source fragments, as well as unexpected isomers may interfere with your analysis. Additionally, as metabolites have different ionization efficiencies in the MS, meaning that an equal signal does not necessarily represent an equal molar abundance in samples, comparisons should only be made between groups of samples for a single metabolite, and within the same analytical run.

A good observation would be something like: “NAD^+^ is twice as abundant in my treated group, when compared to my control group”.

An example of a bad comparison would be: “There is more NAD^+^ in the samples I measured in September, than the samples I measured in a separate run in October.” It is possible for analytical differences over time to account for these observed effects.

Another bad observation would be: “There is more NAD^+^ than NADH in this sample”. It is possible for NAD^+^ and NADH to have the same molar abundance, and still have different intensities/response in the MS.

Keep in mind though, that a *shift* in a ratio can be successfully determined. So it is possible to say: “The ratio of NAD+ to NADH decreases in patients when compared to the control group.” This is because it is possible to see for instance a decrease in NAD^+^ between patients and controls, and an increase in NADH between patients and controls.

## Troubleshooting

Our method is very robust and has been successfully employed by analysts of all experience levels with only minor instructions. Here, some typical difficulties are discussed.

### Problem 1

A common practical error for a technician is to add too much of a solvent to the liquid-liquid extraction. For example, the technician forgets to compensate for the internal standard volume, or adds methanol twice instead of Milli-Q water. This issue can occur during “step-by-step method details step 1”.

### Potential solution

***Note:*** Solvent ratios during the liquid-liquid extractions are important. Methanol and chloroform are miscible, as are methanol and water. It is the 2:1:1 chloroform:methanol:water (v/v/v) ratio that results in a two-phase system. Be aware that because of this, additional chloroform or water in the system is probably not a big issue. However, additional methanol is, as it is this solvent that can dissolve both of the other solvents. Deviations from the protocol may therefore disrupt the two-phase system.

• There is some flexibility in the total volume. If you accidentally add a few microliters too much of a phase, this is unlikely to cause an issue.
• If a double amount of methanol has been added, this would block the possibility of a two-phase extraction. This has happened once in our lab and the solution was to use the vacuum concentrator to evaporate the methanol and start over with the extraction. Samples did not appear to suffer from a double round of exposure to 60°C.

### Problem 2

A metabolite of interest has a high coefficient of variance in the pooled sample analysis, while other metabolites appear to be okay. This issue can occur during “peak integration and statistics step 4”.

### Potential solution

• Check integrations of the peak in all samples. Make sure the correct peak has been integrated in a consistent manner across all samples.
• Check the integrations of the internal standards. Fluctuations in the internal standard can result in large variances. Check other internal standards and check all metabolites corrected for the problematic internal standard to see if they are also affected. It’s possible to correct a metabolite for another, closely related, internal standard in this case.
• Check related metabolites. If GTP is problematic, check if ATP is problematic as well. Focus on chemical similarities and for instance retention time with comparisons. If a class of metabolites is affected, consider maintenance of your instrument if you expect to see this class in your analyzed matrix and their internal standards are also problematic.
• Pooled samples are an average of all your samples and give an idea of how well a metabolite could be measured. However, if a small percentage of samples differs greatly from the others (e.g., high value in controls, low value in patients or vice versa), a metabolite might be easily measured in those high abundance samples, but be too diluted to pick up in the QC successfully. It might still be of interest, precisely because it is so different between groups. First, check if this is the case. If so, the differences can still be meaningful, but realize that calculating values like fold changes will be impossible.
• Check if there is any particular pattern in the QC samples; e.g., a steady decrease in intensity, large enough to explain the high coefficient of variance. If so, see if your analytical instrument requires cleaning or maintenance. Repeat the entire analytical run after maintenance if this is the case.

### Problem 3

The identity of a peak for a metabolite of interest is uncertain, for instance because of interfering peaks or shifted retention times. This issue can occur during “peak integration and statistics step 1”.

### Potential solution

***Note:*** When doubt remains about an identification of a metabolite, it is better to leave the metabolite out of the dataset. It is our experience that uncertainties in identification tend to get lost in communication, especially when data is shared with scientists who are unfamiliar with metabolomics.

***Note:*** It is always best to focus on larger patterns within datasets, rather than individual metabolites. There is always an element of uncertainty and none of these suggestions guarantee a 100% secure identification. If a specific metabolite is important for mechanistic interpretation of the data, we advise to confirm this metabolite abundance with a dedicated targeted metabolomics method.

• If at all possible, spike a sample with a standard of the metabolite of interest. For instance, divide a sample, or the pooled sample, in two, adding an appropriate amount of the standard to one of those halves. Analyze both samples and compare the peaks.
• ZIC-cHILIC peaks can have recognizable shapes; e.g., glucose is always a broad, split peak. This can help aid identification.
• Use MS/MS to fragment peaks of interest; be aware that close isomers will likely have similar fragments and that many metabolites are too small to fragment.
• Use the retention times of internal standards as calibration points for your analytes.
• Use biological knowledge and common sense; e.g., glutathione is one of the most abundant metabolites in cells, and a potential isomer is therefore likely not the largest peak in the sample.
• Sometimes, closely related metabolites cannot be separated, but it is clear what they are. For instance, sugar phosphates, or glycerol phosphates. It has been our choice to report these as a sum of all peaks, under generic names like: “Total sugar phosphates” or “Glycerol-2P_or_3P”.

### Problem 4

A sample shows no, or very limited, peak intensity across the entire metabolome. This issue can occur during “peak integration and statistics step 1”.

### Potential solution

• Compare internal standard intensities to other samples to determine if it is an analytical problem, or a problem with the sample itself. If no endogenous peaks are visible, but the internal standard shows strong signals, then it is a biological problem that cannot be fixed at this time. Possibly too little sample was added and this sample should be considered a loss. If the internal standards show similar issues, continue with point 2.
• Check if the problem concerns mass accuracy. Manually confirm if an internal standard peak is truly absent, or if its mass deviates so far that it is no longer recognized by the integration software. If it’s a mass accuracy issue, check your calibration settings or perform maintenance on the machine.
• Ensure that there aren’t bubbles at the bottom of the sample vial. Surface tension sometimes results in empty space at the bottom of the inserts, resulting in an injection of air. Tap the samples gently if this is the case.
• Reinject the sample only if this is done immediately after the analysis of the entire set of samples. Any changes to the set-up mean that the sample is incomparable to the others due to the relative quantitation this method provides. Either inject the entire sample set again, or remove the sample from the project.

### Problem 5

A sample is a clear outlier, for instance because all intensities are much higher. This issue can occur during “peak integration and statistics step 5”.

### Potential solution

***Note:*** It is out of the scope of this protocol to discuss various outlier tests, or more fundamentally discuss what constitutes an outlier, or when to remove them. This determination is left to the researcher. The goal here is strictly to help understand what might cause outliers when using this method and what steps can be taken to minimize them.

***Note:*** It is important to differentiate between biological and analytical outliers, as these should be treated differently. And, as muscle tissue tends to deal with single samples, as opposed to technical replicates in for instance cell culture experiments, also realize that natural variance is expected.

• Check the peaks of some of the problematic metabolites and their internal standards.
• Check all correction values. First, check if this sample perhaps received too little of the internal standard mixture. This can be done by plotting all of the internal standard peak areas across samples. If this is the case, the sample should be removed from the dataset. Second, check if the value of muscle weight is correct. Alternative corrections, for instance using a Pierce BCA Protein Assay on the protein pellet can also be considered to corroborate dry tissue weight.
• Check if there can be a biological origin for the observed changes. For example, we once encountered heart tissue from a “healthy donor” with towering carnitine abundances, skewing plots. As this appeared to be a true biological outlier, the results for this sample were reported to the collaborator as part of the dataset, with a note mentioning the finding.
• Check for carry-over effects, even though this method typically does not experience many issues in this area. Make sure this sample is not part of a sample group with low metabolite intensities, with this sample being measured after a sample with massive intensities. If this is the case, rerun the analysis, with blanks in between samples.

## Resource availability

### Lead contact

Riekelt H. Houtkooper (r.h.houtkooper@amsterdamumc.nl).

### Materials availability

This study did not generate new unique reagents.

## Data Availability

Metabolomics data from these samples have not been made available here as these data do not belong to the Core Facility Metabolomics. They have been published by Janssens et al. (2022).

## Supplemental information

Table S1. Sample matrix overview, related to Before you begin

Table S2. MS settings, related to step 11 in Step-by-step method details

Table S3. Integration details, related to step 1 in Quantification and statistical analysis
